# Prediction of acute kidney injury after cardiac surgery with fibrinogen-to-albumin ratio: a prospective observational study

**DOI:** 10.3389/fcvm.2024.1336269

**Published:** 2024-02-27

**Authors:** Wang Xu, Xin Ouyang, Yingxin Lin, Xue Lai, Junjiang Zhu, Zeling Chen, Xiaolong Liu, Xinyi Jiang, Chunbo Chen

**Affiliations:** ^1^ Department of Intensive Care Unit of Cardiac Surgery, Guangdong Provincial People’s Hospital (Guangdong Academy of Medical Sciences), Southern Medical University, Guangzhou, Guangdong Province, China; ^2^ Peking University Shenzhen Hospital, Shenzhen, China; ^3^ Day Surgery Center, Affiliated Hospital of Southwest Medical University, Luzhou, China; ^4^ Department of Critical Care Medicine, Shenzhen People’s Hospital, Shenzhen, Guangdong Province, China

**Keywords:** acute kidney injury, fibrinogen-to-albumin ratio, cardiac surgery, biomarkers, cardiac surgery intensive care unit

## Abstract

**Background:**

The occurrence of acute kidney injury (AKI) following cardiac surgery is common and linked to unfavorable consequences while identifying it in its early stages remains a challenge. The aim of this research was to examine whether the fibrinogen-to-albumin ratio (FAR), an innovative inflammation-related risk indicator, has the ability to predict the development of AKI in individuals after cardiac surgery.

**Methods:**

Patients who underwent cardiac surgery from February 2023 to March 2023 and were admitted to the Cardiac Surgery Intensive Care Unit of a tertiary teaching hospital were included in this prospective observational study. AKI was defined according to the KDIGO criteria. To assess the diagnostic value of the FAR in predicting AKI, calculations were performed for the area under the receiver operating characteristic curve (AUC), continuous net reclassification improvement (NRI), and integrated discrimination improvement (IDI).

**Results:**

Of the 260 enrolled patients, 85 developed AKI with an incidence of 32.7%. Based on the multivariate logistic analyses, FAR at admission [odds ratio (OR), 1.197; 95% confidence interval (CI), 1.064–1.347, *p* = 0.003] was an independent risk factor for AKI. The receiver operating characteristic (ROC) curve indicated that FAR on admission was a significant predictor of AKI [AUC, 0.685, 95% CI: 0.616–0.754]. Although the AUC-ROC of the prediction model was not substantially improved by adding FAR, continuous NRI and IDI were significantly improved.

**Conclusions:**

FAR is independently associated with the occurrence of AKI after cardiac surgery and can significantly improve AKI prediction over the clinical prediction model.

## Introduction

Cardiac surgery can lead to a serious complication known as acute kidney injury (AKI), which poses a risk of significant morbidity and mortality (1). The diagnosis and treatment of AKI have historically relied on serum creatinine (SCr) and blood urea nitrogen, as well as urine output. However, clinically significant changes often occur within days of injury, making early treatment and nephroprotective intervention difficult (2, 3). To effectively tackle this problem, it is crucial to promptly detect individuals vulnerable to AKI. This identification will facilitate the implementation of management guidelines suggested by Kidney Disease: Improving Global Outcomes (KDIGO), which comprise adjusting hemodynamics and volume, closely monitoring renal function, and averting nephrotoxicity (4, 5).

Numerous factors have been identified as associated with the risk of postoperative AKI development, including sex, age, diabetes mellitus, surgery type, and perioperative hemodynamic goals (1, 6). Nevertheless, a limited number of investigations have endeavored to evaluate hematological biomarkers as autonomous prognosticators of AKI. Albumin, an indispensable indicator of liver function, has the potential to function as a valuable marker for assessing inflammatory and nutritional status (7). Fibrinogen, a key coagulation protein, is widely recognized as a sensitive indicator of inflammatory status (8). The innovative inflammation-based risk metric, known as the fibrinogen-to-albumin ratio (FAR), has demonstrated its value in predicting adverse outcomes in cancer (9, 10) and cardiovascular disease (11–13). Recent studies has indicated that the levels of preprocedural FAR exhibit a correlation with the incidence of AKI in individuals undergoing emergency percutaneous coronary intervention (14) and elective percutaneous coronary intervention (15) and in children following ventricular septal defect surgery (16). Nevertheless, the association linking the frequency of acute kidney injury (AKI) in individuals who have undergone cardiac surgery and the previously unexplored variable, known as FAR, remains uninvestigated.

Therefore, we undertook a prospective, observational study within the confines of the Cardiac Surgery Intensive Care Unit (CSICU) to assess the efficacy of predictive models based on FAR in the prognosis of AKI after cardiac surgery.

## Materials and methods

### Study design and participants

This prospective observational study was conducted at the Guangdong Provincial People's Hospital. We consecutively enrolled all patients who were admitted to the CSICU after coronary artery bypass graft (CABG), valve, and/or aortic surgery between February 2023 and March 2023. These patients all underwent cardiopulmonary bypass (CPB). Patients were excluded for the following reasons: age under 18, kidney transplantation or nephrectomy, chronic kidney disease (CKD), renal replacement therapy (RRT) prior to CSICU admission, less than 24 h in the cardiac CSICU, or missing clinical data. The primary objective was to identify the occurrence of AKI within one week from the time of admission to the CSICU. The study protocol was approved by the Ethics Committee of Guangdong Provincial People's Hospital (registered approval number: KY2020-103-01).

### Data collection

Overall baseline clinical data were prospectively collected after admission, including age, sex, weight, preexisting medical conditions, smoking history, emergent surgery, American Association of Anesthesiologists (ASA)stage, type of surgery (valve surgery alone, CABG alone, aortic surgery and CABG and valve surgery), baseline SCr, baseline estimated glomerular filtration rate (eGFR), preoperative hemoglobin level, FAR, left ventricular ejection fraction (LVEF), left ventricular end-diastolic dimension (LVDD), norepinephrine use, adrenaline use, dopamine use, and diuretic use. eGFR was calculated based on the CKD Epidemiology Collaboration creatinine equation (17). SCr was measured before the operation, after the operation at CSICU admission, and thereafter at least once daily as part of routine clinical care during CSICU hospitalization. Surgical data included volume of transfused red blood cells (RBCs), plasma, and blood platelets; amount and type of intraoperative fluids administered (crystalloid and artificial colloid); duration of surgery; CPB time; aortic cross-clamping (ACC) time; and intra-aortic balloon pump (IABP) use. IABP implantation data were also collected and sorted. Hemoglobin, hematocrit, and Acute Physiology and Chronic Health Evaluation II (APACHE II) scores after surgery were recorded at CSICU admission. After recovery from anesthesia, the patient's overall condition was assessed using the APACHE II score. We calculated postoperative mean arterial pressure (MAP) as diastolic BP + (systolic BP−diastolic BP)/3.

The resulting variables included the occurrence of AKI within one week of cardiac surgery, renal replacement therapy (RRT), length of stay in the CSICU, duration of mechanical ventilation, use of extracorporeal membrane oxygenation (ECMO), length of hospital stay, and CSICU mortality.

### Definitions

Based on the recent criteria for the diagnosis of acute kidney injury (AKI) associated with cardiac surgery, AKI refers to patients who have undergone cardiac surgery in the preceding seven days and meet the KDIGO standard (18). The KDIGO standard for AKI is characterized by any of the following conditions: a rise of ≥0.3 mg/dl (≥26.5 µmol/L) within 48 h, a rise of ≥1.5 times the serum creatinine (SCr) level within one week, or a urine output of less than 0.5 ml/kg/h within 6 h. The determination of baseline creatinine followed the previously described rules (19) in descending order of preference: the ICU admission considered the most recent pre-ICU value within a range of 30 to 365 days. A stable pre-ICU value >365 days before ICU admission in patients aged <40 years, (stable defined as within 15% of the lowest ICU measurement); a pre-ICU value >365 days before ICU admission and less than the initial SCr at ICU admission; a pre-ICU value (between 3 and 39 days before ICU admission) less than or equal to the initial SCr at ICU admission and not obviously in AKI; the lowest of the initial SCr at ICU admission, the last ICU value, or the minimum value at follow-up to 365 days.

### Statistical analysis

Data analysis was performed using SPSS Version 25.0 (SPSS, IL, USA) and R statistical software (version 4.2.3). To evaluate the continuous variables, we utilized the Wilcoxon rank-sum test. These variables were presented as medians, accompanied by interquartile ranges (P25, P75). For the categorical variables, we used either the chi-squared test or Fisher's exact test for analysis, and these variables were presented as frequencies (percentages). We compared baseline characteristics and hemodynamic parameters between the groups with and without AKI using the methods described above. All tests were two-sided, and a two-sided *p* value < 0.05 was considered statistically significant.

Clinical models were constructed by univariable and multivariable logistic regression. The clinical variables with a *P* < 0.10 in the univariate analysis were included in the multivariate analysis. Multivariate models were built using a forward variable selection method. To assess the added value of biomarkers to the prediction model for postoperative AKI, we developed two logistic regression models. Model 1 consisted of the selected clinical predictors (excluding FAR), while Model 2 incorporated these predictors in addition to the selected biomarkers. In order to assess and compare the efficacy of the mentioned prediction models, the following methodologies were utilized, as described in previous research recommendations: (1) To assess the accuracy of the prediction model, we constructed the receiver operating characteristic (ROC) curve. The accuracy of the model was measured using the area under the ROC (AUC-ROC). To compare the accuracy of different models, we conducted the Delong test (20). (2) In order to compare the prediction performance of the two models, additional measures such as net recognition improvement (NRI) and integrated discrimination improvement (IDI) were used. These measures offer a more comprehensive evaluation of the reclassification concept (21, 22). For subject categories where results were obtained, an upward movement signifies improved classification, whereas a downward movement represents worse classification. The opposite explanation is true for subjects without results. The quantification of the reclassification improvement manifests as the sum of the difference between the proportion of ascending individuals minus the proportion of descending individuals with results and the proportion of descending individuals minus the proportion of ascending individuals without results. This cumulative difference is referred to as the NRI. The central focus of the IDI is the discrepancy between the overall sensitivity and the “1 minus specificity” in the risk model, with and without incorporating novel markers. A higher IDI value indicates the superior predictive ability of the new model. The aforementioned assessment internally validates the predictive performance of Model 1 and Model 2 through the utilization of the guidance technique, replicated 1,000 times.

## Results

### Patient preoperative characteristics

**Figure 1:**
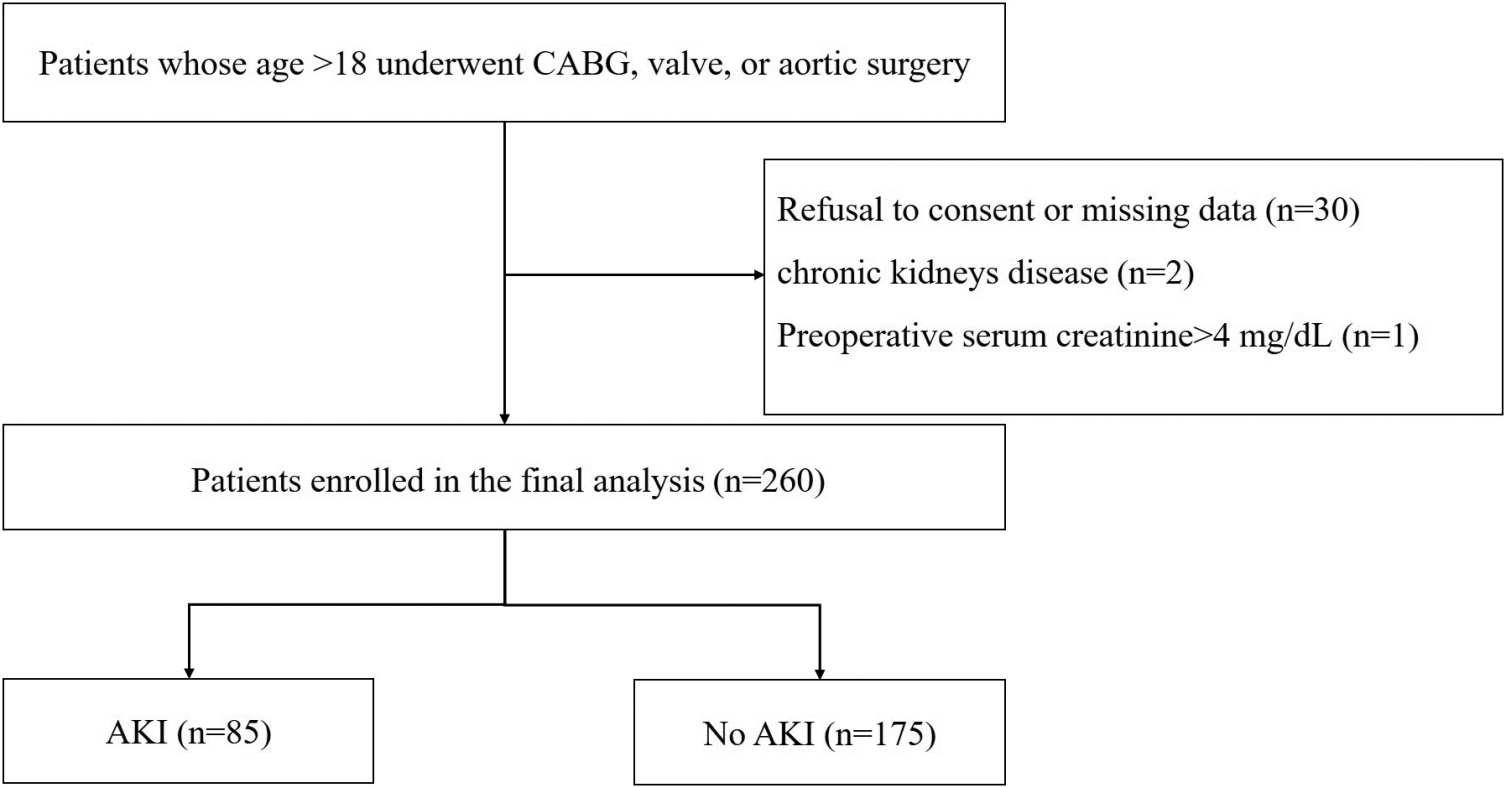
Flow chart from recruitment to outcome. CABG, indicates coronary artery bypass graft; AKI, acute kidney injury.

A total of 293 patients were assessed for eligibility in the research, with 33 individuals being excluded from the study (Figure 1). Subsequently, 260 patients were selected for analysis, and their preoperative characteristics are outlined in Table 1. According to the KDIGO criteria, a total of 85 patients (32.7%) developed AKI within 7 days after cardiac surgery. The percentage of male patients among AKI patients was significantly higher than the percentage of male patients among non-AKI patients. In comparison to non-AKI individuals, patients suffering from AKI had an older age and an increased incidence of emergency surgery. The occurrence of valve surgery and aortic surgery was more prevalent in AKI patients. Additionally, individuals with AKI had elevated baseline SCr, baseline eGFR, hemoglobin levels, and FAR. A notable difference was observed in the administration of norepinephrine and dopamine before surgery in patients with acute kidney injury (AKI) compared to those without AKI. However, there were no significant differences in weight, preexisting medical conditions, smoking history, CABG alone, CABG and valve surgery, ASA classification, baseline eGFR, LVEF, LVDD, adrenaline use or diuretic use between patients with and without AKI.

**Table 1:** Preoperative characteristics of patients with and without postoperative AKI.

Variable	AKI (*n* = 85)	NO AKI (*n* = 175)	*P* value
Demographic variables			
Age, years	60 (50.5,67)	56 (48,61)	0.002
Sex, male, *n* (%)	56 (65.9)	92 (52.6)	0.042
Weight, kg	63 (53.5,71.25)	61 (53,71)	0.507
Preexisting medical conditions, *n* (%)			
Hypertension	30 (35.3)	44 (25.1)	0.089
Diabetes mellitus	6 (7.1)	10 (5.7)	0.672
Coronary artery disease	7 (8.2)	20 (11.4)	0.429
Stroke	8 (9.4)	11 (6.3)	0.364
Heart failure	56 (65.9)	97 (55.4)	0.108
Previous cardiac surgery	6 (7.1)	20 (11.4)	0.271
Hyperlipidemia	4 (4.7)	8 (4.6)	1.000
Smoking history, *n* (%)	22 (25.9)	31 (17.7)	0.125
Type of surgery, *n* (%)			
Valve surgery alone	37 (43.5)	119 (68)	<0.001
CABG alone	14 (16.5)	27 (15.4)	0.829
Aortic surgery	29 (34.1)	19 (10.9)	<0.001
CABG and valve surgery	5 (5.9)	10 (5.7)	1.000
Emergency surgery, *n* (%)	26 (30.6)	8 (4.6)	<0.001
ASA ≥ III grade, *n* (%)	81 (95.3)	167 (95.4)	0.961
Laboratory data			
Baseline serum creatinine, umol/L	82.75 (71.9,100.73)	76.3 (64.67,90.71)	0.011
Baseline eGFR, ml. (min.1.73 m^2^)^−1^	109.23 (84.24,147.2)	104 (73.98,131.42)	0.085
Hemoglobin, g/L	129 (115.5,142)	133 (123,145)	0.022
FAR(%)	9.18 (7.12,12.29)	7.42 (6.35,9.2)	<0.001
Imaging data			
LVEF (%)	64 (58,66.5)	64 (60,66)	0.564
LVDD, mm	48 (43,55)	50 (45,57)	0.155
Medication use, *n* (%)			
Norepinephrine use	20 (23.5)	23 (13.1)	0.034
Adrenaline use	62 (72.9)	130 (74.3)	0.817
Dopamine use	42 (49.4)	61 (34.9)	0.024
Diuretic use	84 (98.8)	171 (97.7)	0.541

CABG Coronary artery bypass grafting; FAR fibrinogen-to-albumin ratio; LVEF Left ventricular ejection fraction; LVDD Left ventricular end-diastolic;.

### Patient intraoperative characteristics

Table 2 shows the intraoperative parameters of AKI and non-AKI patients. In this cohort, it was found that AKI patients received a higher percentage of red blood cells (RBCs) and blood platelets during surgery compared to non-AKI patients. Compared to non-AKI patients, patients with AKI had a longer CPB time and duration of surgery, while there was no significant difference in IABP use. Additionally, patients with AKI received a higher volume of colloid during surgery than patients without AKI.

**Table 2:** Intraoperative data in patients with and without postoperative AKI.

Variable	AKI (*n* = 85)	NO AKI (*n* = 175)	*P* value
Fluid management			
Crystalloid, *n* (%)	15 (17.6)	21 (12)	0.216
Colloid, ml	500 (500,800)	500 (500,1,000)	0.004
RBC, *n* (%)	23 (27.1)	23 (13.1)	0.006
Plasma, *n* (%)	15 (17.6)	17 (9.7)	0.068
Platelets, *n* (%)	37 (43.5)	28 (16)	<0.001
IABP use, *n* (%)	4 (4.7)	7 (4)	0.791
CPB, minutes	196 (146.5,256)	146 (120,188)	<0.001
ACC, minutes	101 (74.5,134)	87 (66,119)	0.041
Duration of surgery, minutes	346 (270,443.5)	260 (218,317)	<0.001

RBC red blood cell; IABP intra-aortic balloon pump; CPB cardiopulmonary bypass time; ACC aortic cross-clamping.

### Patient postoperative characteristics and results

Patients with AKI were likely to have lower hemoglobin and hematocrit levels after surgery on admission to the CSICU and higher APACHE II scores than patients without AKI. The current investigation revealed that the occurrence of AKI was associated with an increased occurrence of postoperative RRT, the use of ECMO, and CSICU mortality. Additionally, patients with AKI had longer hospital and CSICU stays, along with extended periods of postoperative mechanical ventilation (Table 3).

**Table 3:** Postoperative characteristics and outcomes in patients with and without postoperative AKI.

Variable	AKI (*n* = 85)	NO AKI (*n* = 175)	*P* value
APACHE II score	11 (9,13)	7 (6,9)	<0.001
Laboratory data within the first 24 h of ICU admission			
Hematocrit (%)	29.7 (27,33.65)	31.6 (29.3,35.4)	0.002
Hemoglobin, g/L	100 (86,110.85)	106 (97,121)	<0.001
MAP, mmHg	87.33 (80,90)	86.67 (77.33,90)	0.689
Outcome			
RRT during ICU stay, *n* (%)	7 (8.2)	0 (0)	<0.001
Length of ICU stay, days	4 (2,8)	2 (2,3)	<0.001
Length of mechanical ventilation, hours	42 (20.5,95.5)	19 (12,16)	<0.001
ECMO use, *n* (%)	4 (4.7)	0 (0)	0.011
Hospital stay, days	18 (14,24.5)	14 (11,19)	<0.001
ICU mortality, *n* (%)	9 (10.6)	0 (0)	<0.001

MAP, mean arterial pressure; RRT, renal replacement therapy; ECMO, extracorporeal membrane oxygenation.

### Establishment and comparison of models

**Figure 2:**
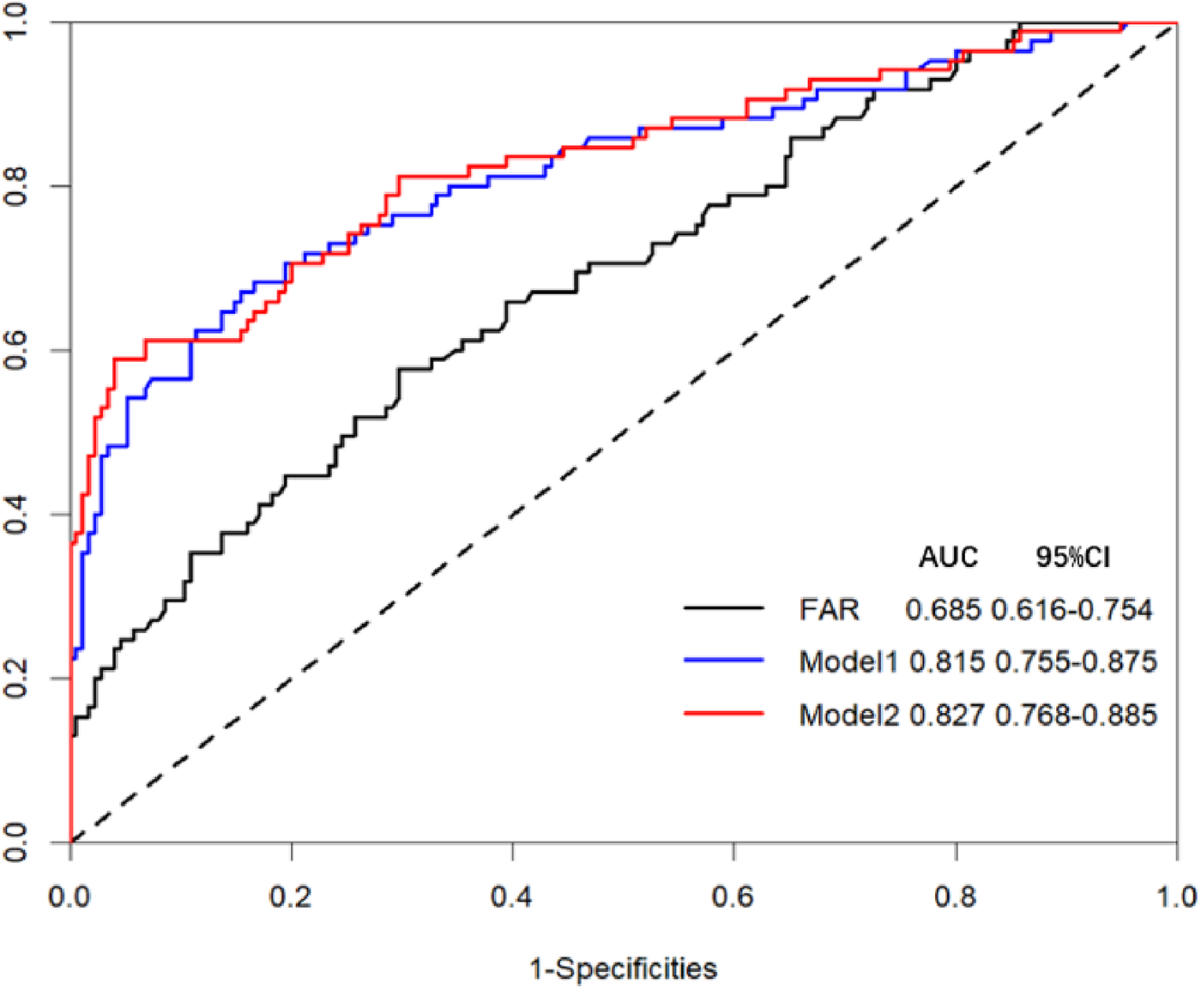
Receiver operating characteristic (ROC) curve analysis for two models and FAR in predicting AKI. Model 1 for AKI prediction is composed of APACHE II, Surgery time, and Aortic surgery; Model 2 for AKI prediction is composed of APACHE II, Surgery time, Aortic surgery, and FAR.

Supplementary Table S1 provides a comprehensive overview of the results obtained from univariate logistic regression analyses. The predictive results are summarized in Table 4, which displays the multivariate regression analysis. The ROC curves of FAR are showcased in Figure 2. Notably, two models were considered: Model 1, which was established based on the identified clinical factors alone, and Model 2, which incorporated FAR in combination with the aforementioned factors. The ROC curve indicated that FAR at admission was a significant predictor of AKI (AUC-ROC = 0.685). Model 1 could reasonably predict postoperative AKI (AUC-ROC = 0.815). The inclusion of FAR improved the AUC-ROC of the prediction model to 0.827, as shown by the ROC curve. Furthermore, the Delong test showed that the difference was not statistically significant (Table 4, *P* = 0.216). However, to ascertain whether Model 2 could improve risk reclassification in comparison to Model 1, both the IDI and the NRI were used. (Table 5). According to the findings, the NRI yielded a value of 0.301 (0.048–0.553), meaning that Model 2 achieved a 30.1% improvement in correct classification compared to Model 1 (*P* = 0.020). Furthermore, the IDI yielded a value of 0.033 (0.009–0.057), indicating that Model 2 showed a 3.3% improvement in overall discrimination ability compared to Model 1 (*P* = 0.008).

**Table 4:** Multivariate logistic regression analysis variables and AUC-ROC analyses of predictive models.

Predictive model and component	OR	95% CI	*P* value	AUC-ROC	95% CI	*P* value^a^
Model1				0.815	0.755–0.875	0.216
APACHE II	1.405	1.251–1.577	<0.001			
Surgery time	1.005	1.002–1.008	0.001			
Aortic surgery	3.013	1.381–6.575	0.006			
Model2				0.827	0.768–0.885	
APACHE II	1.352	1.201–1.521	<0.001			
Surgery time	1.005	1.001–1.008	0.004			
Aortic surgery	2.935	1.303–6.612	0.009			
FAR	1.197	1.064–1.347	0.003			

AUC-ROC, area under the receiver operating characteristic curve; FAR, fibrinogen-to-albumin ratio.

^a^ Versus model 1.

**Table 5:** Net reclassification improvement and integrated discrimination index of two models.

Model	cNRI (95%CI)	*P*-value^a^	IDI (95%CI)	*P*-value^b^
Model1				
Model2	0.301 (0.048–0.553)	0.020	0.033 (0.009–0.057)	0.008

cNRI, net reclassification improvement between two models; IDI, integrated discrimination index between two models; Model 1 for AKI prediction is composed of APACHE II, Surgery time, and aortic surgery; Model 2 for AKI prediction is composed of APACHE II, Surgery time, aortic surgery and FAR.

^a^ cNRI model 2 versus model1.

^b^ IDI model 2 versus model1.

## Discussion

In this single-center prospective study, the occurrence of AKI was frequent, and it was found to be associated with adverse outcomes during hospitalization in the CSICU. The occurrence of AKI after surgery was found to be influenced by several independent risk factors, including FAR, duration of surgery, aortic surgery, and the postoperative APACHE II score. To the best of our understanding, this investigation is the first to reveal that FAR was autonomously correlated with AKI incidence following cardiac surgery and improved AKI prognosis beyond the clinical prediction model. AKI, a frequent complication arising after cardiac surgery, is observed in approximately 5%–42% of individuals worldwide who undergo this procedure annually, totaling over 2 million (1). Our study results demonstrated that in the first week following cardiac surgery, the prevalence of AKI was as high as 32.7% among individuals. Furthermore, the likelihood of developing AKI after cardiac surgery is strongly influenced by the specific type of surgery, resulting in significant differences in incidence rates (23). This conclusion is consistent with our research results. The incidence of AKI may vary between studies due to factors such as variations in patient characteristics (e.g., age groups and types of surgery), differences in sample size, and different definitions of AKI. Several investigations have consistently recognized AKI as an element in prolonged hospitalization, a complicated clinical course, and increased mortality after cardiac surgery (24). However, it is a formidable task to detect and diagnose AKI at an early stage because it presents a wide range of clinical manifestations, spanning from the absence of symptoms to oliguria and potentially even renal failure. Thus, it is crucial to identify clinical characteristics and validate biomarkers that can accurately predict patient prognosis. Achieving such breakthroughs would significantly increase the efficacy of screening and diagnostic tools (25).

During the univariate analysis of this study, it was found that AKI showed a significant correlation with various clinical variables both before and during cardiac surgery. This finding confirmed that AKI can stem from multiple clinical factors. Previous research conducted by Rosner et al. (2) showed that cardiac surgery characteristics, such as the use and duration of CPB and elevated vasopressin levels, were strongly associated with an increased risk of AKI. As per the results of one of our previous studies, patients admitted to the ICU who experienced a postoperative mean arterial pressure (MAP) below 75 mmHg for a duration of one hour or longer were found to have a significant independent association with the occurrence of AKI (26). The multivariate logistic regression analysis of this study also confirmed that aortic surgery and surgery time were risk factors for AKI.

In this study, aortic surgery was identified as an independent risk factor for AKI. Aortic surgery is a complex procedure that requires the use of hypothermic circulatory arrest, which can result in severe renal ischemia-reperfusion injury. While moderate hypothermic circulatory arrest has managed to shorten the duration of CPB in aortic surgery, it is still considerably longer than in other cardiac surgeries. Consequently, aortic surgery further contributes to the risk of developing AKI. Cardiac surgery with CPB is widely acknowledged for its prolonged duration and substantial trauma, frequently resulting in postoperative organ dysfunction in patients (27). Therefore, in our study, surgery time was chosen for multivariate logistic regression. In assessing the risk model, we also considered the APACHE II score, which is a physiologically based system consisting of twelve physiological parameters. Patient SCr levels and chronic kidney function status were among the parameters taken into account. The APACHE II score, a widely recognized prognostic tool, is commonly utilized to predict unfavorable outcomes in ICU patients (6). In the current study, APACHE II was selected as one of the independent predictors in the risk model.

AKI is influenced by inflammation in its pathogenic mechanisms (28). Fibrinogen and albumin are two widely reported proteins with properties related to inflammation, nutrition, and blood flow dynamics (29). Serum albumin plays a crucial role in maintaining colloid osmotic pressure, scavenging free radicals, and altering the permeability of capillary membranes (30, 31). The nutritional and inflammatory status of patients can be effectively assessed by considering the preoperative serum albumin level (32). Lower levels of serum albumin are often correlated with elevated blood viscosity and impaired endothelial cell function (33). Fibrinogen, a glycoprotein primarily synthesized by liver cells, functions as a crucial factor in blood coagulation and the regulation of coagulation pathways (34). In inflammatory situations, there is an upregulation of fibrinogen, which initiates the recruitment of cells involved in inflammation and platelets. Additionally, it activates endothelial cells, contributing to prolonged vascular inflammation, and resulting in platelet aggregation and leakage in the vasculature (35). In various diseases, both fibrinogen and albumin have been identified as important prognostic indicators, according to several studies (36, 37). FAR, an index that combines albumin and fibrinogen, shows higher sensitivity and specificity in predicting systemic inflammation, blood clot formation, and viscosity than fibrinogen and albumin alone (15). Mechanisms of AKI following cardiac surgery include renal reperfusion-induced ischemia, inflammatory response, hemolysis, oxidative damage, and exposure to nephrotoxins (38). We believe that these mechanisms could explain the predictive role of FAR in determining the likelihood of AKI occurrence.

The relationship between FAR and cardiovascular disease has received considerable attention in scientific research. For instance, FAR has shown great potential as a reliable indicator for identifying the exaggerated increase in morning blood pressure in newly diagnosed hypertensive patients who have not yet received treatment (39). Notably, recent studies have uncovered a correlation between elevated levels of FAR and the manifestation of AKI following cardiac surgery. Can Wang et al. discovered a significant independent association between the occurrence of AKI and the preoperative assessment of FAR in patients undergoing percutaneous coronary intervention (15). Fan Cao et al. demonstrated that FAR during CPB was an independent predictor of AKI in infants with ventricular septal defects who underwent cardiac surgery involving CPB (16). However, these studies were limited to children or post-contrast AKI. A difference in our study is that we explored the value of the FAR in predicting AKI after cardiac surgery in adults. Moreover, we included several types of cardiac surgery. Our findings in this study further expand the scope of the application of FAR to AKI after cardiac surgery.

By incorporating FAR into the existing risk model, the model discrimination for AKI showed an increase in AUC from 0.815 to 0.827 (*p* = 0.216), although this difference did not reach statistical significance. It is worth noting that the limited patient sample size may have influenced these findings. Nonetheless, the introduction of FAR into the prediction model yielded a significant improvement in NRI (0.301, *p* = 0.020) and IDI (0.033, *p* = 0.008). The NRI assesses how much patients improve their predicted probabilities, whereas the IDI highlights the average improvement in predicted probabilities (21). In simple terms, 30.1% of patients experienced an improvement in predictability, with an average increase of 0.033 in predicted probabilities when FAR was included in the predictive model. Hence, this study suggests that the inclusion of FAR in a model already containing established risk factors could improve the predictability of AKI. However, in recent times, there have been concerns raised by statisticians about the overestimation of the improvement in predictability of predictive models using the NRI method (40). Despite the widespread use of the NRI in discriminative prediction models in various studies, it is crucial to exercise caution when interpreting these results, and additional evaluation tools should be used to validate our findings.

This study has several limitations. First, the use of data from a single center, the inclusion of a limited number of patients, and a short selection window devalue the statistical calculations, as these calculations may vary in different institutions and patient data sets characterized by varying distributions. Second, laboratory variables were collected before and after CPB without dynamic monitoring. Third, the study cohort was probably heterogeneous in terms of cohort and surgical status, but further studies involving more homogeneous patient samples are needed to confirm our results. Finally, whether any advantage can be gained from FAR modulation (e.g., albumin infusion) in preventing AKI remains to be determined. Additional prospective multicenter studies with larger sample sizes and experimental studies are necessary to verify our results.

## Conclusion

The present investigation confirms that the measurement of FAR before admission to the CSICU has emerged as a distinct risk factor for AKI in patients who have undergone cardiac surgery. This prospective study provides evidence to support the use of FAR assessment as a means of early prediction and subsequent prediction of AKI. Given its convenient accessibility as a biomarker, FAR holds promise for improving patient prognosis in clinical settings.

## Data Availability

The original contributions presented in the study are included in the article/Supplementary Material, further inquiries can be directed to the corresponding author.

## Supplementary material

Supplementary Table S1 Univariate logistic analysis for prediction of AKI.
