# Supplementary material for: Prediction of acute kidney injury after cardiac surgery with fibrinogen-to-albumin ratio: a prospective observational study
Source: Front Cardiovasc Med. 2024 Feb 27;11:1336269. doi: 10.3389/fcvm.2024.1336269 (PMC10927956; doi:10.3389/fcvm.2024.1336269)
Supplement: Supplementary Table S1 — Univariate logistic analysis for prediction of AKI. [file Datasheet1.docx]

**Supplementary Table 1:** Univariate logistic analysis for prediction of AKI

| Variable | OR | 95%CI | P value |
| --- | --- | --- | --- |
| Demographic variables | | | |
| Age | 1.035 | 1.010-1.060 | 0.006 |
| Male | 1.742 | 1.018-2.983 | 0.043 |
| Weight | 1.005 | 0.984-1.027 | 0.629 |
| Preexisting clinical conditions | | | |
| Hypertension | 1.624 | 0.927-2.845 | 0.090 |
| Diabetes mellitus | 1.253 | 0.440-3.571 | 0.673 |
| Coronary artery disease | 0.696 | 0.282-1.715 | 0.430 |
| Stroke | 1.549 | 0.599-4.006 | 0.367 |
| Heart failure | 1.553 | 0.906-2.660 | 0.109 |
| Previous cardiac surgery | 0.589 | 0.227-1.525 | 0.275 |
| Hyperlipidemia | 1.031 | 0.302-3.524 | 0.961 |
| Smoking history | 1.622 | 0.871-3.020 | 0.127 |
| Type of surgery |  |  |  |
| Valve surgery alone | 0.363 | 0.213-0.619 | <0.001 |
| CABG alone | 1.081 | 0.534-2.187 | 0.829 |
| Aorta surgery | 4.252 | 2.211-8.178 | <0.001 |
| CABG and valve surgery | 1.031 | 0.341-3.117 | 0.957 |
| Emergent surgery | 9.199 | 3.947-21.441 | <0.001 |
| Laboratory data | | | |
| Baseline serum creatinine | 1.015 | 1.004-1.026 | 0.007 |
| Baseline eGFR | 1.006 | 1.000-1.011 | 0.039 |
| Hemoglobin | 0.981 | 0.967-0.995 | 0.008 |
| FAR | 1.924 | 1.173-1.427 | <0.001 |
| Imaging data | | | |
| LVEF | 0.980 | 0.949-1.012 | 0.226 |
| LVDD | 0.985 | 0.956-1.015 | 0.329 |
| Laboratory data within the first 24 h after ICU admission | | | |
| APACHE II score | 1.453 | 1.301-1.623 | <0.001 |
| Hematocrit | 0.857 | 0.270-2.722 | 0.794 |
| Hemoglobin | 0.968 | 0.952-0.984 | <0.001 |
| MAP, mmHg | 1.003 | 0.977-1.030 | 0.818 |
| ASA≥III grade | 0.970 | 0.284-3.316 | 0.961 |
| Fluid management | | | |
| Crystalloid | 1.571 | 0.765-3.229 | 0.219 |
| Colloid | 1.001 | 1.000-1.002 | 0.002 |
| RBC | 2.452 | 1.281-4.693 | 0.007 |
| Plasma | 1.992 | 0.942-4.213 | 0.071 |
| Blood platelet | 4.047 | 2.245-7.295 | <0.001 |
| Use of drugs |  |  |  |
| Norepinephrine use | 2.033 | 1.045-3.958 | 0.037 |
| Adrenaline use | 0.933 | 0.519-1.677 | 0.817 |
| Dopamine use | 1.825 | 1.078-3.091 | 0.025 |
| Diuretic use | 1.965 | 0.216-17.855 | 0.549 |
| IABP use | 1.185 | 0.337-4.165 | 0.791 |
| CPB | 1.010 | 1.006-1.015 | <0.001 |
| ACC | 1.005 | 1.000-1.011 | 0.049 |
| Duration of surgery | 1.007 | 1.005-1.010 | <0.001 |

FAR fibrinogen-to-albumin ratio; LVEF Left ventricular ejection fraction; LVDD Left ventricular end-diastolic; MAP Mean arterial pressure; ASA American association of anesthesiologists; RBC red blood cell; IABP intra-aortic balloon pump; ECMO extracorporeal membrane oxygenation; CPB cardiopulmonary bypass time; ACC aortic cross-clamping
